# Novel Visceral-Anastomosis-First Approach in Open Repair of a Ruptured Type 2 Thoracoabdominal Aortic Aneurysm: Causes behind a Mortal Outcome

**DOI:** 10.1155/2013/978625

**Published:** 2013-02-17

**Authors:** Einar Dregelid, Alireza Daryapeyma

**Affiliations:** ^1^Department of Vascular Surgery, Haukeland University Hospital, Jonas Lies Vei 65, 5021 Bergen, Norway; ^2^Department of Vascular Surgery, Karolinska University Hospital, 171 76 Stockholm, Sweden

## Abstract

Case reports to analyze causes and possible prevention of complications in a new setting are important. We present an open repair of a ruptured type 2 thoracoabdominal aortic aneurysm in a 78-year-old man. Lower-body perfusion through a temporary extracorporeal axillobifemoral arterial prosthesis shunt was combined with the use of a branch to the permanent aortic prosthesis to enable rapid visceral revascularization using a visceral-anastomosis-first approach. The patient died due to transfusion-induced capillary leak syndrome and left colon necrosis; the latter was probably caused by a combination of back-bleeding from lumbar arteries causing a steal effect, an accidental shunt obstruction, and hemodynamic instability towards the end of the operation. The visceral-anastomosis-first approach did not contribute to the complications. This approach reduces the time when visceral organs are perfused only via collateral arteries to the time needed for suturing the visceral anastomoses. This may be important when collateral perfusion is marginal.

## 1. Introduction

A subcutaneous axillofemoral bypass has previously been shown to prevent ischemic injury during operations for thoracoabdominal aortic aneurysms [1, 2]. The extracorporeal use of a vascular prosthesis for a temporary shunt has, to our knowledge, only been described in three cases previously [3, 4]. However, the use of a temporary vascular prosthesis shunt has been described without any substantial detail in 10 other cases [5]. A temporary subcutaneous bypass using a vascular prosthesis as opposed to an atrio-arterial bypass eliminates the risk of lower extremity ischemia due to femoral artery cannulation, does not require the same degree of anticoagulation, and causes less activation of blood components [1, 6], but subcutaneous tunneling for the bypass creates a potential bleeding focus and inflicts extra trauma. In the current paper ischemia prevention by lower-body perfusion through a temporary extracorporeal axillobifemoral shunt using a vascular prosthesis was combined with the use of a branch to the permanent aortic prosthesis to enable rapid revascularization of the visceral territories using a visceral-anastomosis-first approach. The patient contracted severe but well-known complications, not specifically associated with this approach: bleeding, massive transfusions, capillary leak syndrome, and colon necrosis with a deadly outcome. Case reports to analyze causes and possible prevention of complications in a new setting are important.

## 2. Case Report

A 78-year-old man, an ex-smoker for the last two years was admitted in April 2009 with acute pain in the lower abdomen and back. He was on current medication for chronic obstructive pulmonary disease and hypertension in addition to statin therapy. He could walk between 1 and 2 flights of stairs before being halted by dyspnoea. He had been evaluated for a thoracoabdominal Crawford type 2 aortic aneurysm 2.5 months before admission. Operation was not recommended due to its high risk. Endovascular treatment with branched grafts or combined with thoracic and visceral debranching was still an experimental procedure without proven superiority compared with open repair [7].

**Figure 1:**
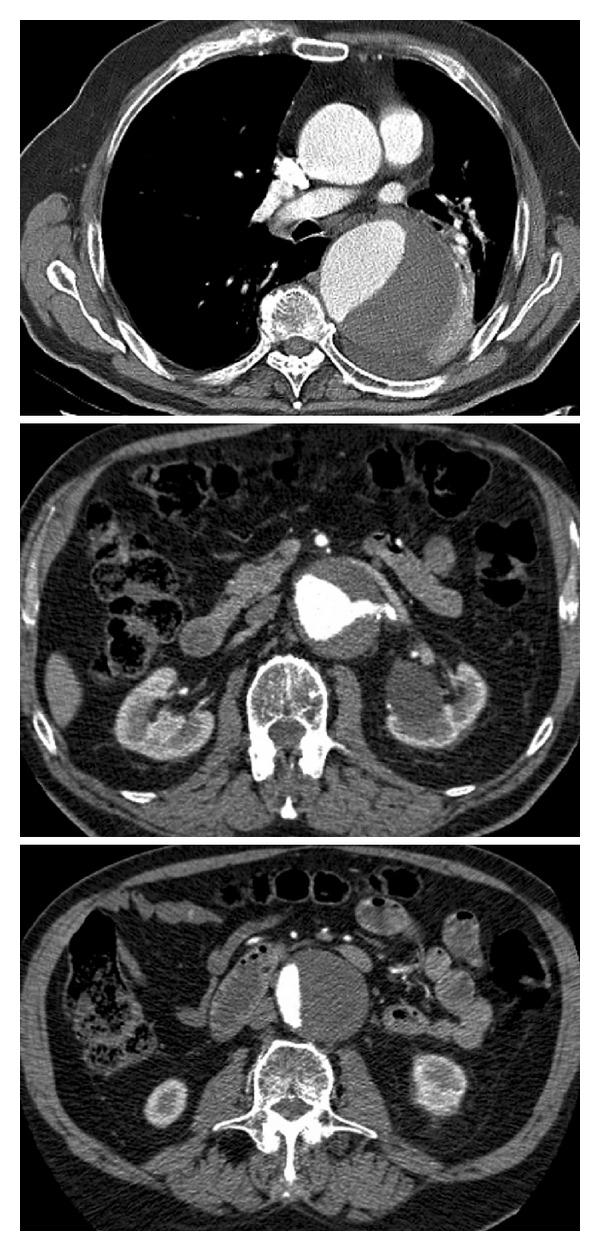
Computed tomography sections at the level of the descending aorta (upper panel), at the level of the left renal ostium (middle panel), and at the level of the infrarenal aorta (lower panel).

On admittance blood pressure was 156/96 mmHg, pulse 81 bpm and regular, and temperature 37.9°C. There was tenderness around the umbilicus. Peripheral circulation and femoral pulses were good. Creatinine was 238 *μ*mol/L, and estimated glomerular filtration rate was 23 mL/min/1.73 m^2^. He was given analgesics and labetalol to lower his blood pressure. Computer tomography showed atelectasis of the lower lobe of the left lung and some pleural fluid. Aneurysm diameter was 8.7 cm in the mid-descending part and 5.5 cm at the level of the renal arteries (Figure 1). Imminent aneurysm rupture was suspected. A few hours after arrival, he developed strong chest and back pain and became hypotensive and oliguric. Haemoglobin fell from 140 to 114 g/L. ECG and troponin analysis showed no sign of myocardial infarction. Rupture of the thoracic part of the aneurysm was suspected. He was informed of poor prognosis both without surgical repair and with the planned procedure. He consented and was operated on urgently.

**Figure 2:**
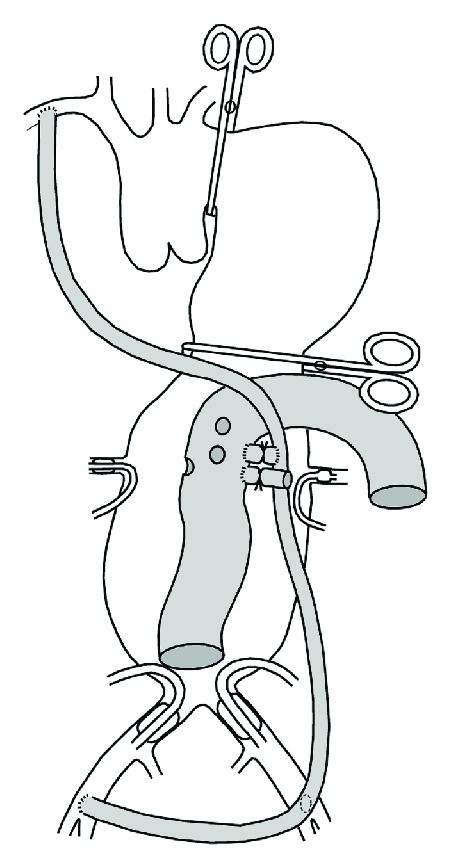
The presutured vascular prosthesis construct (shaded grey) consists of a temporary axillobifemoral bypass with a branch to the permanent aortic prosthesis. The drawing depicts an opened abdominal part of the aneurysm. Two Foley catheters are used for iliac occlusion. The left kidney is perfusion-cooled, and occlusion catheters occlude the right renal (shown) and visceral arteries (not shown). Holes in the middle part of the aortic prosthesis, placed after measurements on preoperative computed tomography images, are ready to be anastomosed to the visceral and right renal ostia. After completion of these anastomoses, the ligature on the connection between the temporary bypass and the aortic prosthesis is removed, and the right kidney and intestines are perfused via the middle part of the aortic prosthesis which is isolated using temporary ligatures (not shown), while a side branch with another temporary ligature is anastomosed to the left renal ostium. Finally, the distal and proximal ends of the aortic prosthesis are anastomosed to the aortic bifurcation and to the aorta just distally to the left subclavian artery, respectively.

With the patient supine, a presutured vascular prosthesis construct (Figure 2), doubly wrapped in tubular drape, was anastomosed to the right axillary and both femoral arteries after heparinization (5000 IU) by two surgeons working simultaneously. All graft components were made of Dacron. Diameters were 10 mm for the axillobifemoral and connection to aortic graft components, 26 mm for the aortic prosthesis, and 6 mm for the side branch to the left renal ostium. A ring-supported prosthesis for the axillobifemoral bypass of adequate size was not available. The vascular prosthesis was clamped close to each anastomosis. The wounds and clamps were doubly draped, and the patient was turned on his right side. Using double lumen intubation, the aorta was exposed using a thoracoretroperitoneal approach with access in the 7th intercostal space.

There was fresh hematoma in the mid-descending aortic wall. Through another thoracotomy in the 3rd intercostal space, the aorta was clamped distal to the left subclavian artery. Another clamp was applied across the aneurysm proximally to the diaphragm. The abdominal part of the aneurysm was opened, 16 Fr Foley catheters were inserted into both iliac arteries for distal control, and axillobifemoral perfusion initiated. Back-bleeding from the celiac, superior mesenteric, and right renal ostium was prevented by occlusion balloons. Cold Ringer acetate solution was infused into the left renal artery. There was copious bleeding from lumbar arteries which were oversewn.

The openings in the aortic graft were anastomosed to the celiac, superior mesenteric, and right renal arteries which were then perfused while the side branch for the left renal ostium was cut short and anastomosed to the latter. Some residual bleeding, initially misjudged to come from an incomplete occlusion by the clamp just proximal to the diaphragm, was deemed to be irremediable. The bleeding subsequently turned out to be from lumbar arteries which were oversewn. After completing the distal anastomosis, the branch between the axillobifemoral shunt and the aortic graft was clamped, and visceral arteries were perfused via the distal part of the aortic graft.

The thoracic part of the aneurysm was opened, and intercostal arteries were oversewn. Access was somewhat hampered by the intact part of the chest wall between the two thoracotomies resulting in additional blood loss. It was now noted that pulses in the graft just proximal to the left groin could no longer be felt. Pulsation in the distal part of the aortic graft was restored by reopening the branch between the axillobifemoral shunt and the aortic prosthesis. After completion of the proximal anastomosis, this branch was removed. The patient developed consumption coagulopathy with airway and stitch-hole bleeding, necessitating multiple extra sutures, repeated haemostatic packing, and altogether 17.4 L of blood products before satisfactory haemostasis was obtained. In addition 5.7 L of salvaged red blood cells with a haematocrit of 40%–50% were reinfused. The thoracoretroperitoneal and thoracotomy wounds were closed; the patient was again turned to the supine position. The subclavicular and inguinal wounds were closed after removal of the axillobifemoral bypass. Oliguria developed toward the end of the operation. A dialysis catheter was placed in the left femoral vein. The operation lasted 15.5 hours. 

Postoperatively, blood gases revealed hypoventilation. Chest X-ray showed no air in the right main bronchus. Ventilation was normalized after retraction of the endotracheal tube. The patient was oedematous and haemodynamically unstable, requiring volume expansion and inotropic support. He subsequently developed a supraventricular tachycardia. Plasma lactate rose to 9.4 mmol/L. Prognosis was considered poor. Life-supportive treatment was stopped, and he died 19 hours postoperatively.

Autopsy showed ischemic gangrene of the left colon. Other organs and lower extremity muscles had been vital until death. There was a horizontal rupture of the mid-descending aortic aneurysm with haemorrhage in the aortic wall and into the mediastinum and 500 mL of bloody fluid in the right pleural cavity. All vessels to the graft were open without any thrombi and with no hematomas around the anastomoses. There were bronchiectases, emphysema, and areas with atelectasis in the lungs.

## 3. Discussion

A visceral-anastomosis-first approach shortens the time when the intestines are perfused only by collaterals fed by the axillo(bi)femoral shunt. This may be important if collateral perfusion is marginal, although Comerota and White obtained good results with an axillofemoral shunt only [1]. In our patient the axillobifemoral shunt provided adequate collateral blood supply to the torso until the visceral vessels had been revascularized as evidenced by a high pressure in the compartmentalized thoracic aorta, copious back-bleeding from lumbar arteries, and a modest blood pressure drop on visceral reperfusion. It cannot be concluded with certainty that the visceral-anastomosis-first approach prevented a more extensive ischemic injury but it reduced the time when viscera were perfused only via collateral arteries to the time needed for suturing the visceral anastomoses and did not contribute to any of the complications that led to the patient's death.

Although in theory the proximal anastomosis could have been performed first during supraceliac aneurysm clamping and perfusion of the viscera with the axillofemoral shunt, the aneurysm was considered too wide to allow safe supraceliac clamping with full arterial pressure in the abdominal compartment of the aneurysm. 

In our patient the right axillary artery was used as an origin for the shunt to allow clamping of the aorta proximally to the left subclavian artery if necessary, and bifemoral perfusion as opposed to unilateral perfusion was elected to optimize collateral supply to torso tissues, to reduce the need for ancillary measures to prevent spinal cord injury and possibly reduce the need for reimplantation of intercostal arteries [8–11]. It turned out that the left axillary artery could have been used in our patient since the aorta could be clamped distally to the left subclavian artery. 

The use of the right axillary artery as an origin for the shunt when the patient lies on the right side demands attention to details and meticulous planning with regard to maintenance of sterility. Reapplication of a vascular clamp on the shunt close to the axillary artery is not straightforward in this position. Taylor et al. constructed an extracorporeal shunt between the left axillary and femoral arteries by suturing two grafts to the arteries and only connecting them after turning the patient on the side. Their approach may make logistics easier with regard to maintenance of sterility [3]. It is important to rehearse the entire operation with the whole surgical and anaesthesia team including scrub nurses. The composition of the team should allow change of personnel in case of exertion during such a long operation.

Hypoperfusion of the left colon may have been caused by back-bleeding from segmental arteries causing a steal effect through visceroparietal collaterals [12, 13], by haemodynamic instability towards the end of the operation, and by accidental obstruction of the axillobifemoral graft just proximally to the left groin. Accidental compression of the axillobifemoral graft can be potentially detectable and preventable by femoral pressure or flow monitoring and by the use of externally ring-supported prosthesis [1]. 

**Figure 3:**
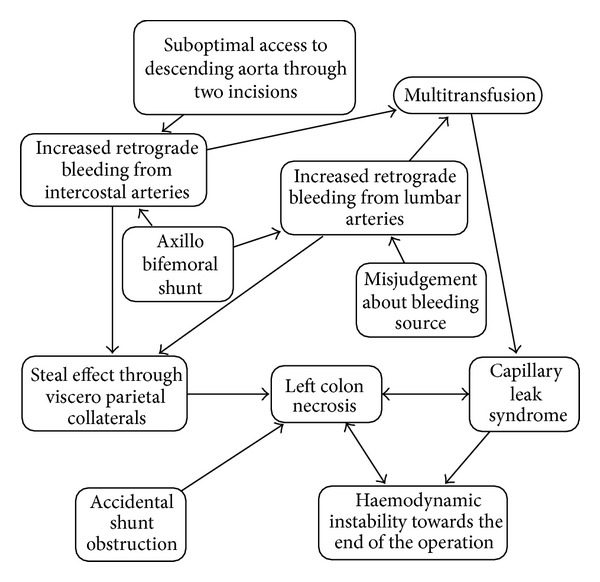
The diagram shows probable causalities of the outcome of the case. The axillobifemoral bypass increases visceral perfusion and retrograde bleeding from lumbar and intercostal branch ostia. Prevention of retrograde bleeding increases visceral perfusion further. Hence insufficient prevention of retrograde bleeding contributes to colon necrosis.

Our patient developed a capillary leak syndrome, attributable jointly to massive transfusion and colon necrosis. The latter might have been detected by opening the peritoneum for bowel inspection before wound closure. Also, bleeding from intercostal arteries might have been reduced using a thoracoretroperitoneal incision to give simultaneous access to the entire descending aorta [11, 14]. In our patient, however, access to the aneurysm through two separate incisions was selected because it was believed that it would confer benefit from the reduced trauma compared with one large incision [15].  Figure 3 shows probable causalities.

Although an axillofemoral vascular prosthetic shunt can be expected to stay open at least for the duration of an operation, despite no pressure gradient [1, 16], competing aortic blood flow exposes the temporary bypass to the risk of thrombosis [17]. An extracorporeal vascular prosthesis shunt as opposed to one buried subcutaneously may be clamped close to the anastomoses until it is needed and for short periods while securing haemostasis [3]. 

## 4. Conclusion 

Visceral and lower limb perfusion via a temporary axillo-femoral or -bifemoral bypass with a branch to the permanent aortic prosthesis using a visceral-anastomoses-first approach may prevent ischemic injury to torso tissues better than only an axillofemoral bypass in operations for thoracoabdominal aortic aneurysms. A meticulously performed haemostasis is essential to avoid consumption coagulopathy and back-bleeding that may cause a steal effect. The colon should be inspected before wound closure after any circulatory instability. Femoral pressure or flow monitoring may allow timely correction of shunt malfunction.
